# Clinical Parasitology and Parasitome Maps as Old and New Tools to Improve Clinical Microbiomics

**DOI:** 10.3390/pathogens10121550

**Published:** 2021-11-28

**Authors:** Stefania Pane, Maria Vittoria Ristori, Simone Gardini, Alessandra Russo, Federica Del Chierico, Lorenza Putignani

**Affiliations:** 1 Microbiology and Diagnostic Immunology Unit, Unit of Microbiomics, Bambino Gesù Children’s Hospital, IRCCS, 00146 Rome, Italy; stefania.pane@opbg.net (S.P.); mvittoria.ristori@opbg.net (M.V.R.); alessandra.russo@opbg.net (A.R.); 2 Multimodal Laboratory Medicine Research Area, Unit of Human Microbiome, Bambino Gesù Children’s Hospital, IRCCS, 00146 Rome, Italy; federica.delchierico@opbg.net; 3 GenomeUp, Via Nemorense 91, 00199 Rome, Italy; simone@genomeup.com

**Keywords:** parasites, microbiota, Faecal Microbiota Transplantation (FMT)

## Abstract

A growing body of evidence shows that dysbiotic gut microbiota may correlate with a wide range of disorders; hence, the clinical use of microbiota maps and fecal microbiota transplantation (FMT) can be exploited in the clinic of some infectious diseases. Through direct or indirect ecological and functional competition, FMT may stimulate decolonization of pathogens or opportunistic pathogens, modulating immune response and colonic inflammation, and restoring intestinal homeostasis, which reduces host damage. Herein, we discuss how diagnostic parasitology may contribute to designing clinical metagenomic pipelines and FMT programs, especially in pediatric subjects. The consequences of more specialized diagnostics in the context of gut microbiota communities may improve the clinical parasitology and extend its applications to the prevention and treatment of several communicable and even noncommunicable disorders.

## 1. Introduction

The intestinal environment is an ecosystem where biological and chemical interactions occur at various organizational levels between host, parasites, and microbial communities, greatly affecting human health and physiology.

Considering the gut microbiota at the taxonomic level, we can observe a significant variation among individuals, each harboring a unique collection of bacterial species, which may change over time and could be considered a fingerprint [1,2,3].

The microbiota at the gut microenvironment level provides important protective, immune regulatory and metabolic functions. The defensive mechanism against pathogenic bacteria is exerted by the barrier effect of the intestinal epithelium, playing a major role in protecting the host and representing an important obstacle to pathogenic invasion [4,5]. Indeed, gut microbiota has an important role in immunological activation and development, as demonstrated in many metabolic and autoimmune diseases [6], by influencing host immune response [7,8,9,10,11]. The balance of the gut microbial ecosystem, eubiosis, is an important concept. Indeed, the eubiotic gut microbiota is characterized by a preponderance of potentially beneficial species, belonging mainly to the two bacterial phylum Firmicutes and Bacteroidetes, and, potentially, pathogenic species such as Proteobacteria. In the dysbiosis state, “bad bacteria” predominate “good bacteria” [12,13,14].

Moreover, environmental factors, such as age, diet, stress, drugs, and infections including parasitosis, strongly influence the composition of the human microbiota [15,16,17,18]. Nowadays, it is evident that the intestinal microbiota has an important impact on human pathophysiology, also regulating pathogenic burden [19]. Intestinal helminths may directly influence the immune system through their effects on both gut luminal and mucosal microbiota [20]. Indeed, Th2 cells have an important role in combatting parasitic infections [21]. However, in existing literature, there is conflicting evidence on the effect of parasites on microbiota ecology and function and their impact on health and disease balance.

Within this complex scenario, intestinal citizens (e.g., viruses, mycetes, and parasites) interact with the microbial community, modifying the balance between host and gut microbiota [4,22]. The intestinal microenvironment, considered as a whole community, provides an important protective mucosal defense mechanism, but there is evidence that change in the composition of the commensal microbiota alters the gut environment, making this composition vulnerable to pathogenic organisms [23,24,25]. Many factors such as antibiotics, psychological stress, physical stress, modern diet, and hygiene can affect microbial stability, and thus contribute to intestinal dysbiosis [13].

## 2. Role and Relationship amongst Gut Microbiota Citizens

In addition to bacteria, other key microorganisms, such as yeasts and filamentous fungi, viruses, and phages, are present in the gut [26,27]. Moreover, for ~25% of the world’s population, the gut microbiota also comprises intestinal protozoan and worms, namely meiofauna [28,29].

**Figure 1:**
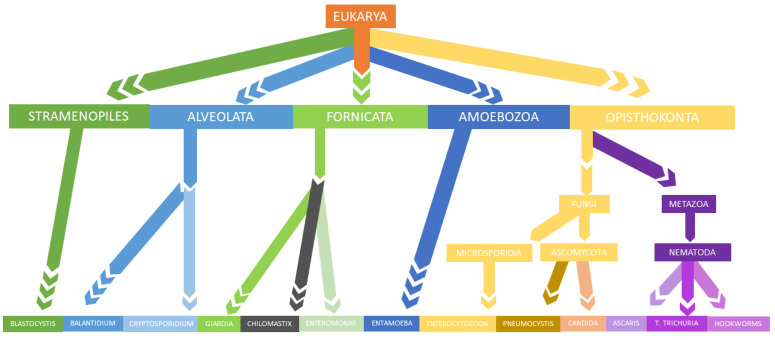
Simplified taxonomic overview of the meiofauna of the human GI tract.

The diversity of meiofauna living on or in our bodies is associated to all metazoans with dimensions between 30 µm and 1 mm. Many members of the meiofauna significantly affect morbidity and mortality, including fungi (e.g., *Candida, Aspergillus*), unicellular protozoa (e.g., *Giardia, Entamoeba*), and helminthic worms (e.g., *Ascaris*) (Figure 1) [30].

Sequencing technologies allow us to analyze the global diversity of the meiofauna of the human gastrointestinal tract to intimately demonstrate gastrointestinal meiofauna may be important in promoting health or disease. Several studies indicate that diet can influence the proportions of the meiofauna and that there is the possibility of trans-kingdom interactions in the gastrointestinal tract [30] (Figure 1).

All these microorganisms offer additional dimensions to the investigation of the host microorganism and microorganism–microorganism interactions. These interplays can be exploited only by combined models obtained by metagenomics, metabolomics and metaproteomics harmonization and integration [31], aiming at producing decision support systems for disease stratification in medicine [32].

All microbial inhabitants may have both beneficial and detrimental roles in the human health, including improvement of microbial resilience, immune evasion, maintenance of physiologic processes, but even alteration of microbial communities [33]. Bacteriophages in the gut are largely unexplored, despite their potential to regulate bacterial communities and thus human health [34,35]. Indeed, experimental limitations still successfully affect the isolation of phages and genome annotation as well as the full characterization of virus–human populations [36]. Some studies have highlighted the uniqueness of phage communities in individuals and their capacity to be stable in a healthy gut [37]. Remarkably, the stability of the viral genome is probably responsible for the stableness of bacteria and microbiota metagenomes [38]. However, there are still only a few studies on the role of the virome in the host’s intestinal microbiota ecosystem [39].

Fungi are normal inhabitants of the mammalian gastrointestinal tract. In fact, the human gut is colonized by more than 50 genera of fungi [40]. In particular, the gut is characterized by the presence of *Candida*, *Saccharomyces* and *Cladosporium* species. Nutritional modification may have an effect on the fungal microbiota; in particular, plant-based diets increase the levels of *Candida*, whereas animal-based diets improve the presence of *Penicillium* species [41,42]. Indeed, fungi compose a very small portion of gut microbiota but play determinative roles in the homeostasis of the gut bacterial composition and the mucosal immune responses. An interkingdom correlation between bacteria and fungi has been suggested. Alterations in the composition and function of the gut microbiota are a usual event in patients who suffer from IBD. Although the main reason for this alteration is not clear, the interaction between gut bacteria and gut fungi seems to be an important subject in IBD patients [43].

So, intestinal parasites, both protozoans and metazoan (nematodes and platyhelminths), interact with the microbial community, modifying the balance between host and gut microbiota [44].

## 3. Parasites and Gut Microbiota Profiling

Parasitic infections represent a significant health problem, particularly in underdeveloped and developing countries. Soil-transmitted helminths (STHs), e.g., *Strongyloides stercoralis* and *Trichuris trichiura*, are common intestinal parasites, followed by blood flukes (i.e., *Schistosoma* spp.) and filarial worms (e.g., *Wuchereria* and *Brugia*). Generally speaking, intestinal parasitic infections may have a low impact in immunocompetent subjects; however, they can potentially become a major issue in vulnerable groups. In fact, helminth infections produce malnutrition, physical damage, and cognitive development complications in children [45]. Furthermore, a wide range of protozoans are common parasites of the human gastro-intestinal tract (e.g., *Cryptosporidium* spp., *Entamoeba histolytica*, *Giardia duodenalis*). The spectrum of clinical manifestations of protozoan infections varies from chronic diarrhea or weight loss, or mild self-limiting illness to acute disease, until malabsorption [29,46,47], as well as the modality of transmission, zoonotic or anthroponotic, particularly investigated as reported for Apicomplexan parasites [48]. A study conducted on packaged salads highlighted the presence of protozoan contamination, such as *Giardia duodenalis*, *Cryptosporidium* spp., *Toxoplasma gondii*, and *Cyclospora cayetanensis*, through microscopy and molecular analyses, concluding that 4.2% of the samples were contaminated by at least one protozoan species, and 0.6% of samples with at least two protozoa [49].

Together with trillions of microorganisms, i.e., archaea, viruses, bacteria, and eukaryotes residing in the GI tract, parasitic worms establish the “macrobiota” [50]. The microbiota of a host may strongly interfere with the survival and physiology of many parasites and, consequently, with the outcome of many parasitic infections. During parasite infections, the interaction between the gut microbiota and the helminths has been associated with the establishment of the infection, the clinical manifestations, and even immune modulation [51,52]. However, both increased and decreased gut microbiota diversity has been observed in subjects affected by intestinal parasites [53,54,55,56,57].

Experiments performed in murine models of intestinal schistosomiasis have suggested interactions between *Schistosoma* parasites and the host gut microbiota with a direct impact on the intestinal microbial communities [58]. In a study by Alba Cortés et al., the gut microbiome composition of the host actually influenced the host’s susceptibility to *S. mansoni* infection, as well as infection-associated changes in gut microbiota profiles [58]. Some studies have evidenced that *Necator americanus* infection could alleviate chronic inflammation in celiac disease and improve prokaryotic species richness, reestablishing the eubiosis and immune homeostasis [59]. In a case report on *Strongiloides stercoralis* infection, gut microbiota composition was associated with enrichment in *Bifidobacterium*, *Blautia*, *Ruminococcus*, *Bacteroides*, *Corynebacterium*, *Colinsella*, *Streptococcus*, *Coprococcus*, and *Oscillospora* genera, and a decrease in *Staphylococcus*, *Lactobacillus*, and *Pediococcus*. The authors suggested a putative direct or immune-mediated ability of *S. stercoralis* to promote the increase in bacterial diversity [60]. In some studies conducted in a rural African population, the authors showed a characteristic gut microbiota ecology in patients carrying *Entamoeba* colonization [61,62,63] (Table 1).

A review of the literature tried to study in depth the mutual influences of intestinal nematodes and host-gut microbiota, highlighting the potential beneficial effects (i.e., promotion of eubiosis) through the production of useful metabolites (i.e., short-chain fatty acids, SCFA) [60]. Conversely, nematode infection may promote dysbiosis due to promotion of pathogenic bacterial species and decrease in mutualistic commensal. Authors exemplified the nematode–microbiota interactions and their impact on the host immune response [64].

A shotgun metagenomics study on samples of patients with the *Blastocystis* spp. infection showed a very strong association between the presence of *Blastocystis* spp. and the abundance of archaeal organisms (*Methanobrevibacter smithii*) [65]. Additionally, another study showed a decrease in *Blastocystis* in individuals with the *Bacteroides* enterotype compared to subjects with the *Ruminococcus* or *Prevotella* enterotypes [66]. Audebert et al. highlighted a high microbiota diversity, increased abundance of Clostridia, and low abundance of Enterobacteriaceae in *Blastocystis*-colonized patients, suggesting that *Blastocystis* infection may be associated with a healthy gut microbiota [67]. Conversely, another study did not highlight significant modifications in the gut microbiota of *Blastocystis positive* subjects affected by irritable bowel syndrome [68]. In a rat model of *Blastocystis* ST3 infection, the colonization altered gut microbiota composition, but not richness, inducing only mild gut inflammation but no clinical symptoms. In addition, the long-term *Blastocystis* exposure appeared to promote faster recovery from colitis, suggesting that *Blastocystis* may alter the gut ecosystem in a protective way and promote faster recovery [69]. Conversely, in healthy subjects, the fermentation by anaerobic bacteria and *Blastocystis* induced an increased SCFA production [70]. A further study has developed a bioinformatic pipeline to detect *Blastocystis* subtypes (STs) from shotgun metagenomics data, identifying *Blastocystis* as a common component of the healthy gut microbiome [65]. The authors showed how metagenomics could play an important role in advancing evidence on population genomics of human parasites. Therefore, through DNA sequencing-based methods, it might be possible to address the role of *Blastocystis* and *Dientamoeba* as commensal components of healthy gut microbiota rather than pathogenic microorganisms.

In other papers, *B. hominis* and *D. fragilis* appeared to be more or less obligated eukaryotic members of the gut microbiota [71,72,73] and less frequent in subjects with intestinal diseases and metabolic disorders [65,74,75,76].

Conversely, other studies have highlighted the possible role of *D. fragilis* and *B. hominis* as infectious agents, providing a relationship with gut microbiota profiles and host phenotypic features, including microbiota dysbiosis or eubiosis [70]. Indeed, in this article, Stensvold C.R. et al. discussed the possible role of *Blastocystis* as an indicator of disease-related gut microbiota profiles in subjects affected by gut dysbiosis [70] or as an indicator of eubiosis in healthy subjects.

Since the composition of the intestinal bacterial population affects the impact of the infection of protozoans, or vice versa, the modulation of different components of the microbiota could be used to prevent or attenuate intestinal protozoan infection and the ultimate outcome of parasitic disease (Table 1). Indeed, microbial signatures associated with parasitic infections may represent the actual link with the gut microbiota shape, playing a role in unveiling the susceptibility to infections and in their clinical outcome in terms of disease onset, progression, and severity [77].

**Table 1:** Gut microbiota profiles associated to parasitic infections.

Reference	Parasite	Type of Infection	Type of Study	Type of Sequencing	Gut Microbiota Composition
[67]	*Blastocystis*	Natural	Human study	16S rDNA sequencing	Increase in Clostridia, Mollicutes, Clostridiales, Ruminococcaceae and Prevotellaceae. Decrease in *Bacilli,* Lactobacillales, Enterococcaceae, Streptococcaceae, Lactobacillaceae and Enterobacteriaceae.
[68]	*Blastocystis*	Natural	Human study	16S rDNA sequencing	No significant change.
[69]	*Blastocystis*	Mouse infected with Blastocystis ST3	Murine model	16S rDNA sequencing	Increase in *Bilophila* and *Butyricimonas* in the *Blastocystis*-colonized group. Decrease in Defluviitaleaceae.
[65]	*Blastocystis*	Natural	Human study	Shotgun metagenomics	Increase in Firmicutes and Clostridiales. Decrease in *Bacteroides*.
[61]	*Entamoeba*	Natural	Human study	16S rDNA sequencing	Increase in Bacteroidales, Mollicutes, Christensenellaceae, Elusimicrobiaceae, Ruminococcaceae, Paraprevotellaceae, *Treponema*, *Parabacteroides*, *Streptococcus*, *Butyrivibrio*, *Oscillospira*, *Desulfovibrio* and *Ruminococcus bromii*. Decrease in *Prevotella*, *Prevotella copri.*
[62]	*Entamoeba*	Natural	Human study	16S rDNA sequencing	Positive correlation between *Bifidobacterium* vs. *B. fragilis*, and *Prevotella* vs. *Bacteroides*. Negative correlation between *Bifidobacterium* vs. *Bacteroides.*
[63]	*Entamoeba*	Culture of *E. histolytica*	Culture study	16S rDNA sequencing	Increase Lactobacillaceae, Clostridiaceae, Erysipelotrichaceae, and Bifidobacteriaceae.
[58]	*Schistosoma*	Infected with *S. mansoni* cercariae (Sm-exp)	Murine model	16S rDNA sequencing	The authors supposed that susceptibility to *Schistosoma* infection in mice is partially dependent on the composition of the host baseline microbiota.
[59]	*Necator americanus*	Percutaneous infection with third-stage larvae *N. americanus*	Longitudinal study	16S rDNA sequencing	Increase in Tenericutes, Mollicutes and *Parabacteroides*.
[60]	*Strongiloides stercoralis*	Natural	Case report	16S rDNA sequencing	Increase in *Bifidobacterium*, *Blautia, Ruminococcus*, *Bacteroides*, *Corynebacterium*, *Colinsella*, *Streptococcus*, *Coprococcus*, and *Oscillospora*. Decrease in *Staphylococcus*, *Lactobacillus*, and *Pediococcus.*

## 4. New Molecular Approaches in Translational and Clinical Parasitology

In January 2019, the Parasite Microbiome Project (PMP) began to understand the role of parasite-associated gut microbiota in the pathophysiology of helminthiases [78]. The PMP tried to draw best practices for experimental studies to ensure reliable comparisons between data sets and the introduction of appropriate controls to identify possible environmental microbial contaminants [78]. In a recent review, the authors suggested four elements that must be considered when the scientist wants to generate reliable and reproducible data [50]. As a first step, they propose generating appropriate negative controls (“blanks”) in each step of the experiment, followed by the microscopy-based visualization of helminth-associated bacteria to identify and characterize worm microbiomes across different helminth tissues and developmental stages. Best practices are becoming more and more important because clinical metagenomic next-generation sequencing (mNGS) is rapidly moving from research to clinical laboratories. Chiu et al. focused on the challenges of implementing mNGS in the clinical laboratory and addressing potential solutions for maximizing its impact on patient care and public health [79]. In fact, metagenomic methods (NGS) are the new approach that microbiologists and parasitologists should undertake to improve accuracy and sensitivity, with respect to the classical approach based on the microscopical method, which may be affected by ambiguities and subjective interpretations [80]. Indeed, major usage of different genomic approaches, such as metataxonomics and metagenomes [81], may assist in reducing ambiguities and subjective interpretation in parasite description and assessing their relationship with microbial communities. Marchesi et al. suggested metataxonomics to assess fine metataxonomic tree descriptions of the entire microbiota and metagenomics to assemble microbial genomes and characterize new genes after annotation through shotgun sequencing [81]. Particularly, metataxonomics refers to targeted sequencing of 16S rRNA gene hypervariable regions [82] andallows representative bacterial taxonomic description [83].

In this context, remarkable is the choice of primers able to reduce potential bias in the representation of entire taxonomic units [84,85,86]. The sequencing output represents a set of clusters of close related sequences, called operational taxonomic units (OTUs) [87]. Through bioinformatic OTUs analysis, it is possible to assess the level of microbial community diversity, both in terms of evenness and richness [88] and the degree of divergence between different ecosystems or sample types [89].

On the contrary, shotgun metagenomics is based on DNA molecules, which are randomly broken into fragments that are then sequenced [90]. Hence, the shotgun metagenomic approach provides major information on the taxonomic composition and inferred functional genes, thus fully characterizing the sample, despite requested high coverage [91].

**Figure 2:**
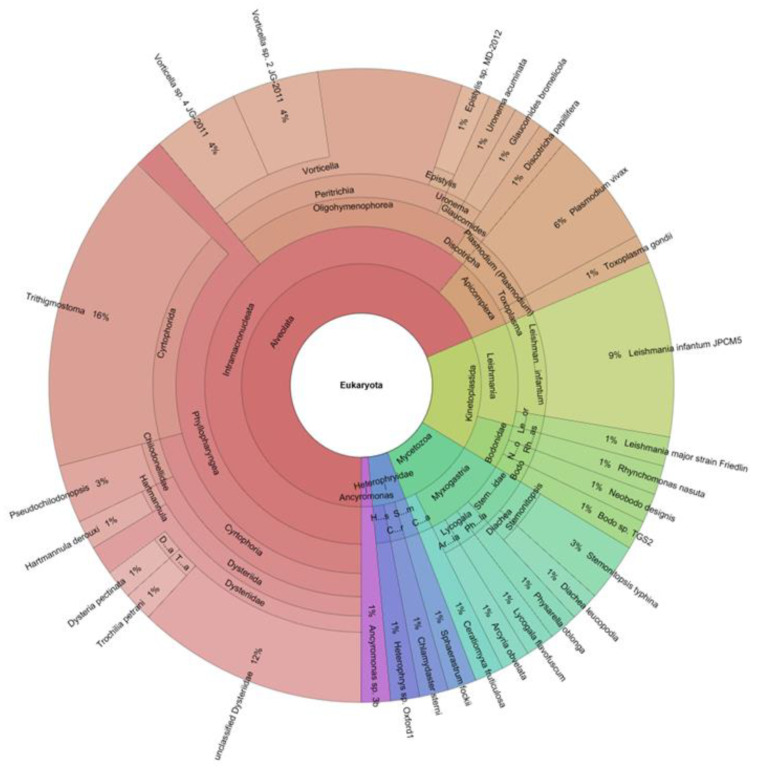
An example of gut parasitome kronos graph obtained by applying agnostic metagenomics pipelines and referred to Eukaryota superkingdom.

**Figure 3:**
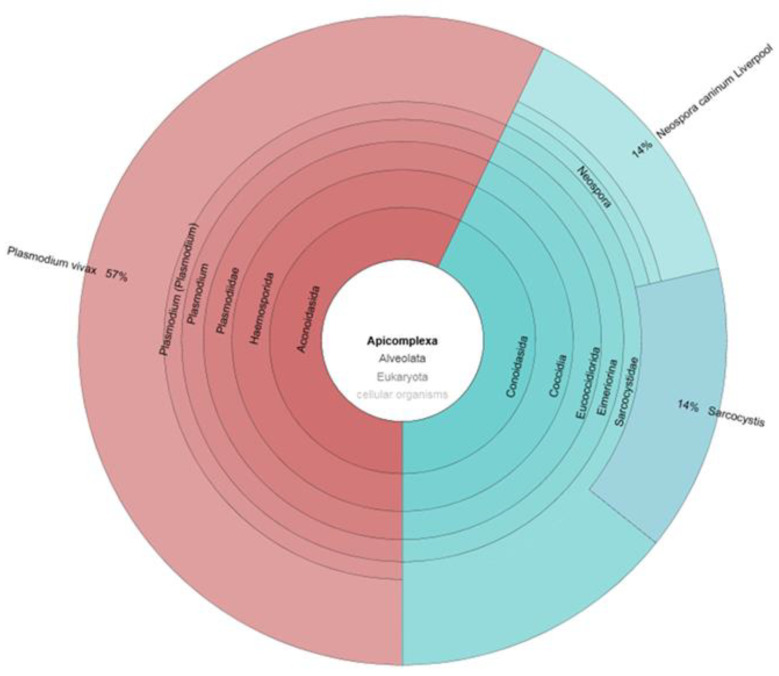
An example of gut parasitome kronos graph obtained by applying agnostic metagenomics pipelines and referred to Apicomplexa phylum.

Clinical applications of metagenomic sequencing include direct identification of microorganisms from primary clinical samples, antimicrobial resistance prediction by characterization of resistance genes, detection of species-level or strain-level virulence determinants, and antiviral resistance prediction. Furthermore, by means of NGS techniques, it is now possible to analyze the onset and progression of infectious diseases in acute and chronic stages [79]. The current challenge will become the agnostic approach through the complete characterization of enteric microbial communities, shallow metagenomics, and trans-kingdom metagenomics (Figure 2 and Figure 3).

## 5. Gut Microbiota Profiling as Tools to Restore and Modulate Gut Microbiota

Gut dysbiosis may be harmful to human beings, leading to organ-localized inflammation, delocalized low inflammation, neuro-inflammation, and mucosal tissue damage predisposed to pathological conditions, such as in the case of obesity, liver steatosis, autism, and PANDAs [92,93,94,95]. Regarding inflammatory mechanisms, amongst infectious diseases, the infection by *C. difficile*, exerted by the toxin causing gastrointestinal illness, is associated with a wide spectrum of severity, ranging from mild diarrhea to pseudomembranous colitis, toxic megacolon, sepsis, and death 17 [96,97]. *C. difficile,* a Gram-positive spore-forming bacillus, is considered a member of the normal gut microbiota. Regardless, its abnormal growth is suppressed by other more dominant anaerobes [98]. The gut colonization of *C. difficile* is reversely related with host age, growing in early infancy and senescence, and decreasing in adulthood. Furthermore, gut colonization of *C. difficile* depends on the loss of the commensal microbiota barrier, loss potentiated by antimicrobial therapies.

**Figure 4:**
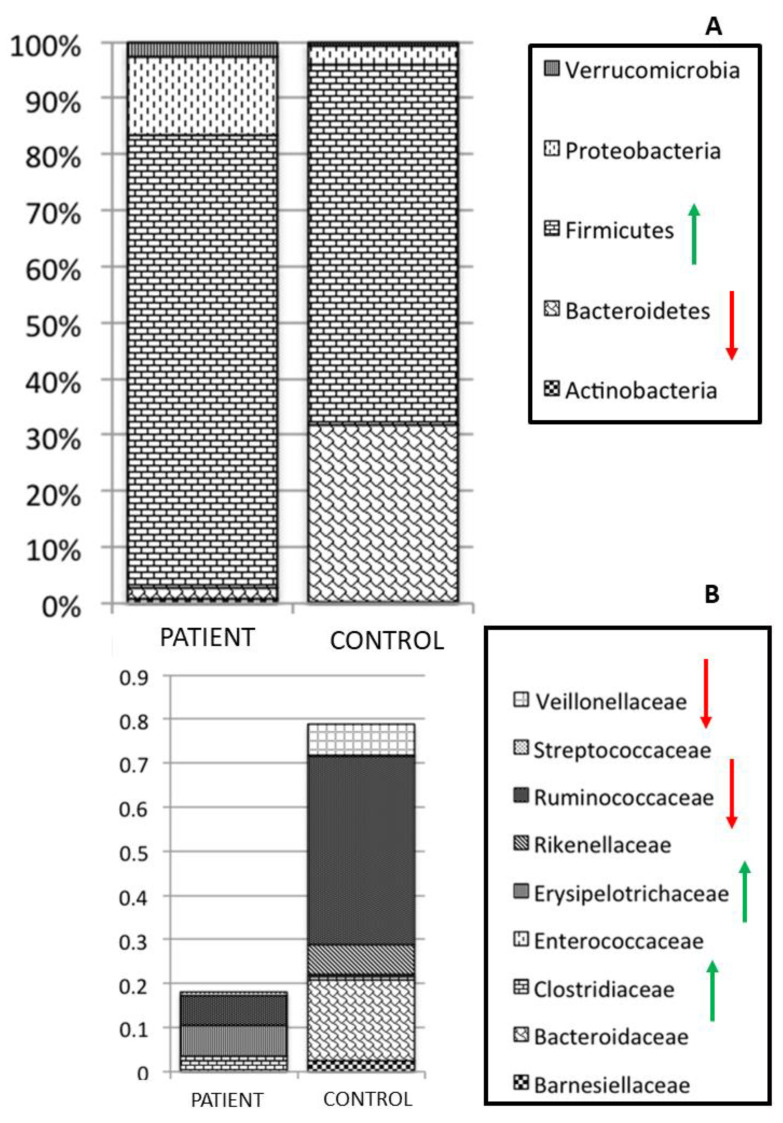
Faecal microbiota map of a patient affected by a *Clostridium difficile* infection. Panel (**A**,**B**) describe global distribution of the microbiota map at Phylum and Family taxonomic levels. Red and green arrows refer to the respective decrease and increase in microbial signatures associated with the disease-driven profile.

In this context, *C. difficile* infection is responsible for the initiation of the cascade of inflammatory processes, which may play an important and destructive role in the initiation and perpetuation of intestinal inflammation [98]. Patients with recurrent *C. difficile* infections are characterized by almost monomicrobial bacterial distributions of the fecal microbiota [99]. A decrease in bacterial diversity and a strong variation in global distribution of OTUs are, indeed, registered in the fecal microbiota profiling of adult patients who present *C. difficile* infections [99]. In particular, a statistically significant increase in Firmicutes and a decrease in Bacteroidetes phyla, compared to healthy subjects are observed in the patients, as well as an increase in Clostridiaceae and Erysipelotrichaceae families (Figure 4). Remarkably, in microbiota profiles associated with infectious agents, the dysbiosis index (DI) is usually very high, based on the prevalence of few microbial taxa, as reported by diagnostic maps obtained for *C. difficile* infection, for which observed dysbiosis values are actually very high (DI > 35%) (https://www.ospedalebambinogesu.it/parassitologia-98785/, (accessed on 20 September 2021)) (Lorenza Putignani and Antonio Gasbarrini, oral communication, 24°Congresso Nazionale delle Malattie Digestive, Rome, FISMAD 23 March 2018) (Patent IT: PCT40659, EU: PCT/IT2017/000119, *Metagenomic Method for* in vitro *diagnosis of intestinal disbiosis*).

Fecal microbiota transplantation (FMT) has achieved a major role in the clinical management of *C. difficile* infections. In these cases, the transplantation is followed by a re-establishment of diversity, and, in many cases, the percentage of efficacy is greater than 90% [100]. FMT is considered as the “ultimate probiotics” because it directly changes the intestinal microbial composition of the host, thus restoring eubiosis and intestinal homeostasis. FMT donor screening is a key factor in the safety of the procedure in order to prevent iatrogenic infectious diseases that are potentially transmittable to the recipient [101]. In fact, the international consensus on stool banking for FMT has recently established that donor stool must be tested for protozoa and helminths, including *B. hominis*, *D. fragilis*, *G. duodenalis*, *Cryptosporidium* spp., *Isospora,* and *Microsporidia* [102]. In addition, the donor must be negative in blood nematodes testing (i.e., *S. stercoralis*) [102], and particular attention is necessary for the parasitological screening of donors when recipients are children [103]. Remarkably, the fecal material (i.e., emulsion) can be stored frozen in a stool bank for use when needed [103]. Indeed, FMT requires a combination of expertise and appropriate methods to identify the best donors, including advanced clinical parasitology.

## 6. Materials and Methods

Regarding the literature review, this section of the paper was conducted to analyze the role of microorganisms (e.g., bacteria, viruses, fungi, worms, and protozoa) on the microbial communities of the gastrointestinal tract both under conditions of eubiosis and dysbiosis. The research was conducted on PubMed, using the following terms: “virus” “fungi”, “worms”, “protozoa”, “microbiota” or “microbiome”, “dysbiosis” or “eubiosis”. Principal articles providing sufficient information about the relationship between the gut microbiota, NGS, clinical parasitology, and FMT were considered. The inclusion criteria for the study were as follows: (1) observational prospective and retrospective studies, case-control studies, cohort studies, or systemic review; (2) studies investigating gut microbiota profiles and parasites infection; (3) studies written in English. All the studies that did not fall in the stated criteria were excluded from the reviewing process. Regarding gut microbiota map generations, 16S targeted metagenomics maps were reported for single-case microbiota profiling under clinical microbiomics settings and in standardized and strict quality procedures. According to OPBG diagnostic procedures, three fecal samples for each subject were collected, processed to optimize microbial DNA extraction, amplified to generate 16S rRNA libraries, pooled, quantified, and sequenced [104] by a MiSeq DX platform, in accordance with the manufacturer (Illumina, CA, USA). In addition to the wet part of the map generation pipeline, the dry bioinformatics module was assessed by an automated process designed by OPBG-GenomeUP in accordance with the OPBG patent. (Patent IT: PCT40659, EU: PCT/IT2017/000119, *Metagenomic Method for* in vitro *diagnosis of intestinal disbiosis*). Regarding shotgun procedures, before DNA extraction, differential sample homogenization (Precellys 24 original tissue homogenizer, (Bertin Instruments, Montigny-le-Bretonneux, France) was performed to optimize parasite and bacteria DNA extraction. Hence, AllPrep Power DNA/RNA Kit was exploited to provide shotgun DNA library templates (bacteriome, parasitome). Library preparation was set up by using Illumina^®^ DNA Prep, (M) Tagmentation (96 Samples), IDT^®^ for Illumina^®^ DNA UD Indexes Set A, NextSeq 550 High Output Kit v2.5 (300 Cycles) according to Illumina specific instructions. Sequencing was performed on the NextSeq550 platform, ensuring at least a sequencing depth of 50–100 M reads/sample. For the dry protocol, a pre-processing procedure was assured by the removal of the input sequences containing regions of low quality/complexity and readings of less than 50 nucleotides. Only reads passing QC filters were directed to the following steps, in which data were mapped against the human genome. In the third step, a comparison with reference databases was undertaken, and taxonomic annotation was provided by processing GenBank and RefSeq databases. Alignments were filtered based on identity percentage and query coverage. Reports were generated as CSV files, HTML interactive tables, and Krona graphs [105]. Taxonomic and functional annotation were processed by Python statistics modules.

## 7. Conclusions

Over the last two decades, helminths and protozoans, previously considered only as pathogens, have been being increasingly suggested as also commensal, protective, or even curative microorganisms. These findings essentially point out that most of these primarily considered “parasites” could have an evolutionary history, tipping the balance towards commensalism, where they have adapted to live off the host without causing any harm. However, it is important to keep in mind that for most of these data, there are other studies that have found opposing results. A likely explanation for these differences can be attributed to various parameters affecting the study design and protocols. The method of analysis, which includes the type of sample used for sequencing, the workflow standardization, and lab-to-lab or operator variability, can create important biases in the results. Moreover, the majority of the studies rely on fecal samples, which does not give enough information on the localization of the parasite within the GI tract. The characterization of the human gut parasitome is now urgent, but this still requires time and new tools. The “-omics” analyses, i.e., (meta)genomics, (meta)transcriptomics, (meta)proteomics, (meta)metabolomics), represent a robust tool for such types of studies [106]. In this context, the application of “big data” methodologies to GM may boost this intervention. The introduction of sequencing technologies has revolutionized the field, enabling investigators to characterize microbial communities. By utilizing larger datasets, researchers are able to design large-scale studies to ask (and answer) complex questions. Metadata associated with samples are becoming an increasingly large contributor to microbiome big data and the challenges associated with streamlining data analysis. The successful application of big microbiome dataset analysis has already provided relevant insights for other areas of research, such as epidemiology, agriculture, and healthcare. Since gut microbiota composition differs widely according to host genetics, diet, lifestyle, geographical location, and disease burden, the application of big data methodologies to gut microbiota and parasitome profiling could be of utmost importance in developing a wide understanding of various infectious diseases. The reach of a new type of clinical parasitology, encountering the approaches and needs of the clinical microbiomics [107], may contribute to the new data-driven era in medicine, establishing new connections with other big data and genome-wide association links, opening the way to a novel holobiont perspective in infectious diseases.

## Data Availability

Not applicable.
